# Automated Vertebral Segmentation and Measurement of Vertebral Compression Ratio Based on Deep Learning in X-Ray Images

**DOI:** 10.1007/s10278-021-00471-0

**Published:** 2021-07-08

**Authors:** Dong Hyun Kim, Jin Gyo Jeong, Young Jae Kim, Kwang Gi Kim, Ji Young Jeon

**Affiliations:** 1 Department of Medicine, Gachon University College of Medicine, Gil Medical Center, 38-13 Docjeom-ro 3beon-gil, Namdong-gu, Incheon, 21565 Republic of Korea; 2 Department of Health Sciences and Technology, GAIHST, Gachon University, Incheon, 21999 Republic of Korea; 3 Department of Radiology, Gil Medical Center, Gachon University College of Medicine, 21 Namdong-daero 774beon-gil, Namdong-gu, Incheon, 21565 Republic of Korea

**Keywords:** Deep learning, Segmentation, Vertebral compression fracture, Vertebral compression ratio

## Abstract

Vertebral compression fracture is a deformity of vertebral bodies found on lateral spine images. To diagnose vertebral compression fracture, accurate measurement of vertebral compression ratio is required. Therefore, rapid and accurate segmentation of vertebra is important for measuring the vertebral compression ratio. In this study, we used 339 data of lateral thoracic and lumbar vertebra images for training and testing a deep learning model for segmentation. The result of segmentation by the model was compared with the manual measurement, which is performed by a specialist. As a result, the average sensitivity of the dataset was 0.937, specificity was 0.995, accuracy was 0.992, and dice similarity coefficient was 0.929, area under the curve of receiver operating characteristic curve was 0.987, and the precision recall curve was 0.916. The result of correlation analysis shows no statistical difference between the manually measured vertebral compression ratio and the vertebral compression ratio using the data segmented by the model in which the correlation coefficient was 0.929. In addition, the Bland–Altman plot shows good equivalence in which VCR values are in the area within average ± 1.96. In conclusion, vertebra segmentation based on deep learning is expected to be helpful for the measurement of vertebral compression ratio.

## Introduction

Vertebral compression fractures (VCFs), a deformity of vertebral bodies found on lateral spine imaging, are most commonly seen in osteoporosis [1, 2].The clinical diagnosis of the VCF is determined by the patient presenting with back pain, followed by the spinal images with a fracture in the body of the thoracolumbar or lumbar vertebra [3]. Several imaging modalities are available for evaluation of vertebral compression fractures (VCFs). Since X-ray, consisting of anteroposterior and lateral views of the vertebrae, is the initial diagnostic modality for vertebral compression fractures as it is the fastest and most accessible imaging modality in clinical practice. Another imaging modality used to evaluate VCFs is computed tomography (CT) scan. CT scans are primarily used for areas where plain films suggest there may be injury. They can help detect complex fractures and occult bony injuries not readily apparent on X-ray. It also allows concomitant assessment of cranial, thoracic, and abdominal visceral injuries. MRI is helpful for better visualization of spinal cord compression and ligamentous disruption. MRI is also useful in evaluating the age of the VCFs and in differentiating benign osteoporotic fractures from malignant fractures. Therefore, MRI mainly serves as a problem-solving modality to determine the age and etiology of VCFs [2, 4, 5]. Among the VCFs, the osteoporotic VCF is typical, and percutaneous vertebroplasty is the main treatment [6]. Vertebral compression ratio (VCR) is a typical index in diagnosing VCF [7]. The concept of VCR is a ratio of abnormal to normal anterior vertebral height (AVH), and it is a standard for the diagnosis with spinal disorders such as a scoliosis or VCF [8]. However, it is hard to determine the standard of VCR because the measurement of deformity is variable depending on the scan region, way of measuring, and deformity of the vertebral body before abnormal condition [9–11]. There have been studies on how different methods to measure VCR are related to spinal disorders.

An accurate segmentation in spinal images is essential to measure VCR. However, it is not only labor-intensive for the spine specialists to manually segment the images, but they may also produce the differences in radiographic images [12]. As the deep learning model developments are recently in progress and the fast, accurate segmentation become widely available, the specialists can save time with the automatic segmentation models and produce more consistent images.

Segmentation on spinal radiographic images is currently in continuous progress; however, studies on how the segmented data are clinically used have not been in progress yet. In this study, we segmented the lateral vertebral images using deep learning and produced an algorithm measuring VCR based on the segmented vertebral data.

## Related Work

There have been lots of approaches to find an efficient measurement of VCR. Said sadiqi et al. (2016) investigated the frequency of the VCR measurement methods currently used by surveying 279 spine specialists from different countries [13]. The two most commonly used techniques are the methods comparing AVH and PVH and the average ratio of AVH and adjacent AVH. It showed that the former is more frequently employed than the latter as 51.3–56.8(%), 32.4–40.6(%) in cervical bones, 44.2–66.7(%), 25.9–39.3(%) in thoracic bones, 40.4–66.7(%), and 25.9–42.9(%) in thoracolumbar bones, respectively, were observed. However, Wei-En Hsu et al. (2019) studied about different parameters for measuring the collapse of the vertebral body in VCF [8]. Using four parameters which are VCR, percentage of anterior height compression (PAHC), percentage of middle height compression (PMHC), and kyphotic angle (KA), they assessed vertebral body collapse. The result was that VCR was higher than the PAHC (− 2.5% to 27.74%). According to the study, VCR may be a rapid and simple method for vertebral body height loss assessment, but if the collapse occurs in both anterior and posterior wall, the degree of vertebral body height loss can be underestimated, and PAHC is expected to be the accurate method for examining the collapse of the vertebral body.

Also, as the interest of deep learning used in clinical research increased, studies about vertebral segmentation using deep learning are increasing as well [14]. Nikolas Lessmann et al. (2019) proposed an automated segmentation model with fully convolutional network (FCN) [15]. Fifteen normal thoracolumbar CT scans, 10 normal lumbar CT scans, and 15 lumbar CT scans, 55 low-dose chest CT scans, and 23 T2-weighted MRI scans were employed. This resulted in the dice similarity coefficient of 96.3% in thoracolumbar CT scans, 94.6% in lumbar CT scans with VCF, 93.1% in low-dose chest CT scans, 96.5% in normal lumbar CT scans, and 94.4% in lumbar MRI. Kim et al. (2019) attempted to segment lumbar images with M-net model to evaluate the VCF [16]. The X-ray of 797 patients was employed to train the model, leading to the dice similarity coefficient of 91.60 ± 2.22. According to this study, as an increase of the cases of osteoporosis is occurring due to the aging society, quick and accurate diagnosis of the VCF is necessary, and corresponding development of an automatic vertebral segmentation model is essential. Anjany et al. (2018) suggested a novel method based on deep learning for the segmentation of the spine CT images [17]. Attention-Net was used for localization, and Segmentation-Net was used for segmentation which was trained with random sample 3D overlapping patches from the input volumes. The result of segmentation was 87.60 ± 5.0 of dice similarity coefficient, which is not high comparing with the state-of-the-art models. Although the study got a low percentage of dice similarity coefficient, it is meaningful that their approaches show more accurate segmentation in degenerated cases.

## Method

### Development Environment

In this study, MATLAB was utilized for image processing on pre- and post-processing of the data of the study. The systems for deep learning training consist of four NVIDIA RTX 2080Ti graphics processing units and 128 GB of RAM. The deep learning development environment was done through Python 3.6.9 and Keras 2.2.4 framework at Ubuntu 14.04 operating system.

### Datasets

In this study, X-ray image data of 339 patients with spine disorders in Gachon Gil Hospital (IRB Number: GDIRB2019-137) were collected. These collected data were composed of images in the format of 16-bit Digital Imaging and Communications in Medicine (DICOM) and were converted to 8-bit images in this study. This dataset has a total of 339 X-ray images containing 205 images as training data, 67 images as validation data, and 67 images as test data. Also, the model was evaluated with result data manually segmented by the spine specialists. All images were de-identified before inclusion in this study.

### Pre-processing

**Fig. 1:**
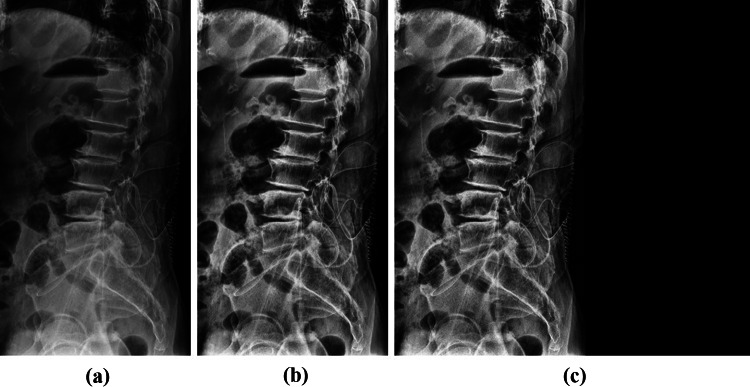
Image processing and zero paddings images of the vertebra. **a** Original image, **b** CLAHE and Gaussian filter, **c** Padding image

Due to the narrow range of intensity distribution of X-ray data, the image contrasts were enhanced by distributing intensity values after applying the contrast-limited adaptive histogram equalization (CLAHE) [18]. The Gaussian filter was applied to the images with enhanced contrast to remove the noise. When resizing images, pixel spacing information is missed. So we reduced the image size according to the aspect ratio and applied zero paddings to produce the 512 × 512 image. Figure 1 indicates the original X-ray and pre-processed images.

### Multi Dilated Recurrent Residual U-Net

**Fig. 2:**
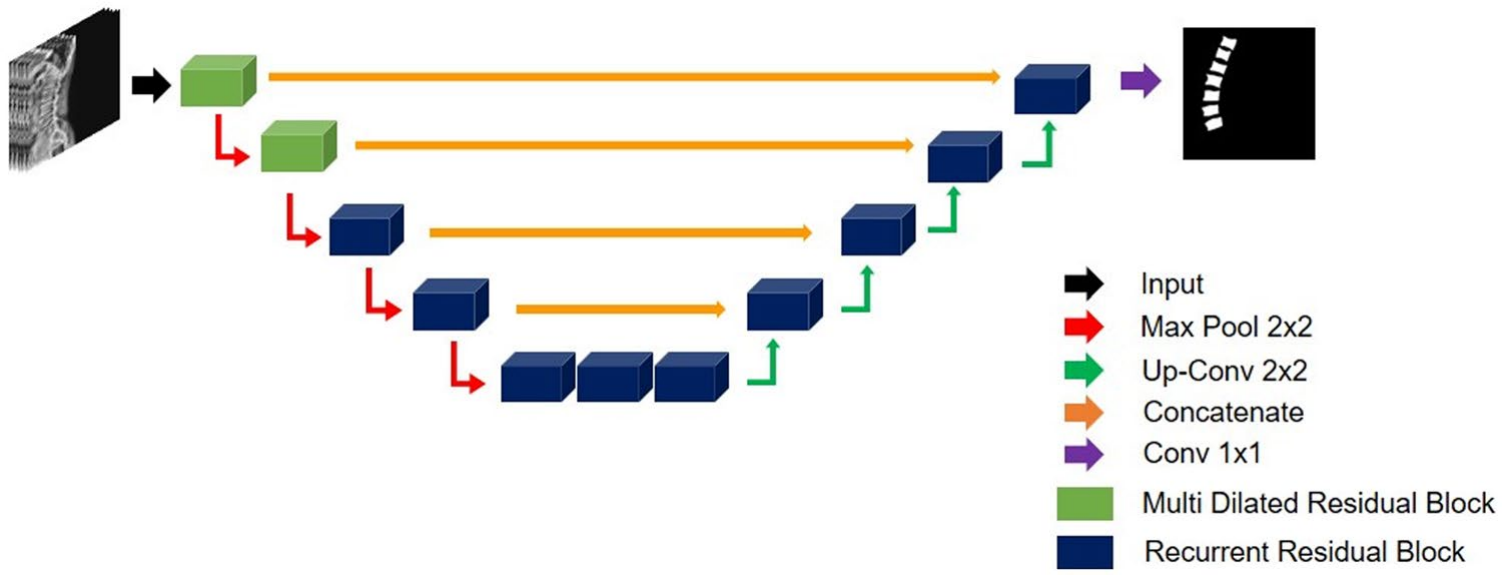
Multi dilated recurrent residual U-Net architecture

The proposed multi dilated recurrent residual U-Net (MDR2-UNet) is shown in Fig. 2, which consists of Multi Dilated Residual Block (MDRB) and Recurrent Residual Block (RRB). First, MDRB is used as a feature encoder to extract features from various receptive fields at the start of the model. MDRB includes dilate convolution, batch normalization, and ReLU and consists of a bottleneck layer that concatenates feature maps through four dilate convolutions to reduce train parameters. This will be explained in detail in the “Multi Dilated Residual Block” section. Second, in the RRB, the residual unit enables training in the deeper model, and the recurrent unit is used to improve the expression of features through feature accumulation. Using these two blocks, the segmentation performance was improved. The orange arrow in Fig. 2 compensates for the loss of localization information through convolution by concatenating the feature map before max pooling in each block. 1 × 1 convolution, the last layer of the model, was used for the binary classification.

### Dilated Convolution

**Fig. 3:**
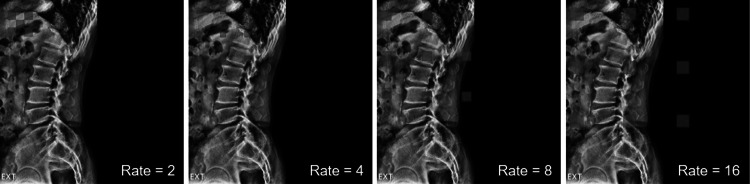
Illustrations of a dilated convolution

Dilated convolution is a method of increasing the receptive field by adding zero-padding inside the filter. The receptive field is the area where the filter was viewed at once, and the higher the receptive field, the more useful it is to extract features from the image. The dilated convolution is used when the receptive field needs to be viewed broadly or when the GPU memory is insufficient due to the large kernel size convolution. Dilated convolution can have a large receptive field without pooling, so the spatial dimension loss is small. Also, since the weight, except the value calculated through dilated convolution, is 0, the computational efficiency is good. It is mainly used in segmentation tasks because it maintains the spatial features. Figure 3 indicates dilated convolution.

### Multi Dilated Residual Block

**Fig. 4:**
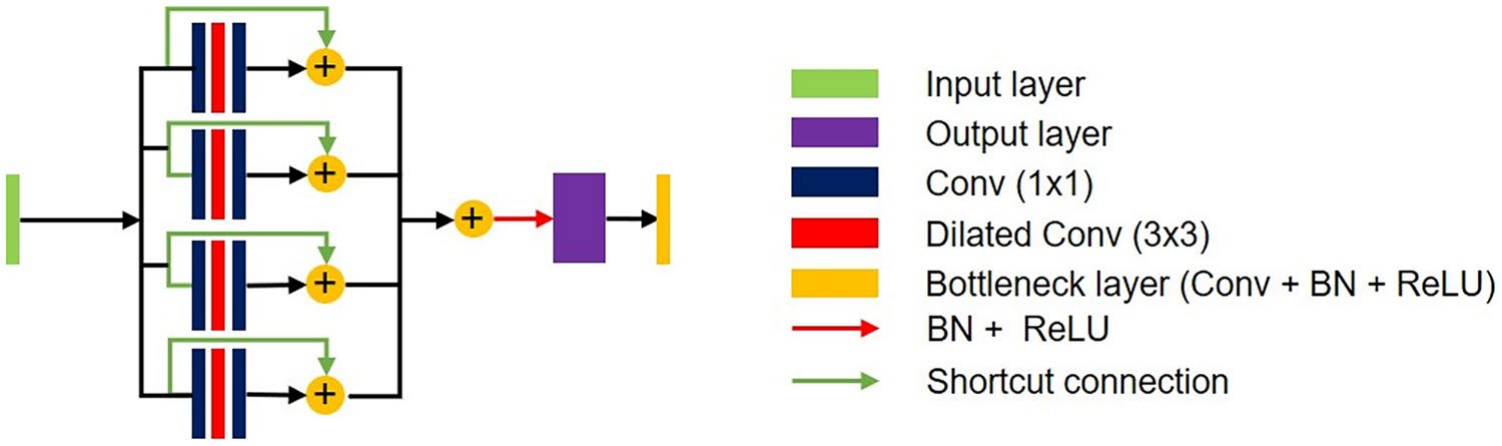
Multi dilated residual block

Multi dilated residual block is a residual block using shortcut connection between four dilated conversion layers with different dilate rates and input layers. There are several advantages for the proposed block in segmentation tasks. First, all feature maps were considered for training by concatenating feature maps extracted from various receptive fields using different dilated rates in the dilated convolution layer. When the feature map is concatenated, the dimension of the feature map becomes quadruple, and the train parameter increases, which increases the amount of computation. To solve this problem, MDRB includes a bottleneck layer. It reduces the feature map dimension and the train parameters. Multi-scale dilated convolution layers contain four dilated rates, rate = 2, 4, 8, and 16. Second, MDRB applied shortcut connection between the input layer and output layer of dilate convolution. It helps to train between the input layer and output layer of dilated convolution in the deeper model. Figure 4 indicates MDRB.

### Recurrent Residual Block

**Fig. 5:**
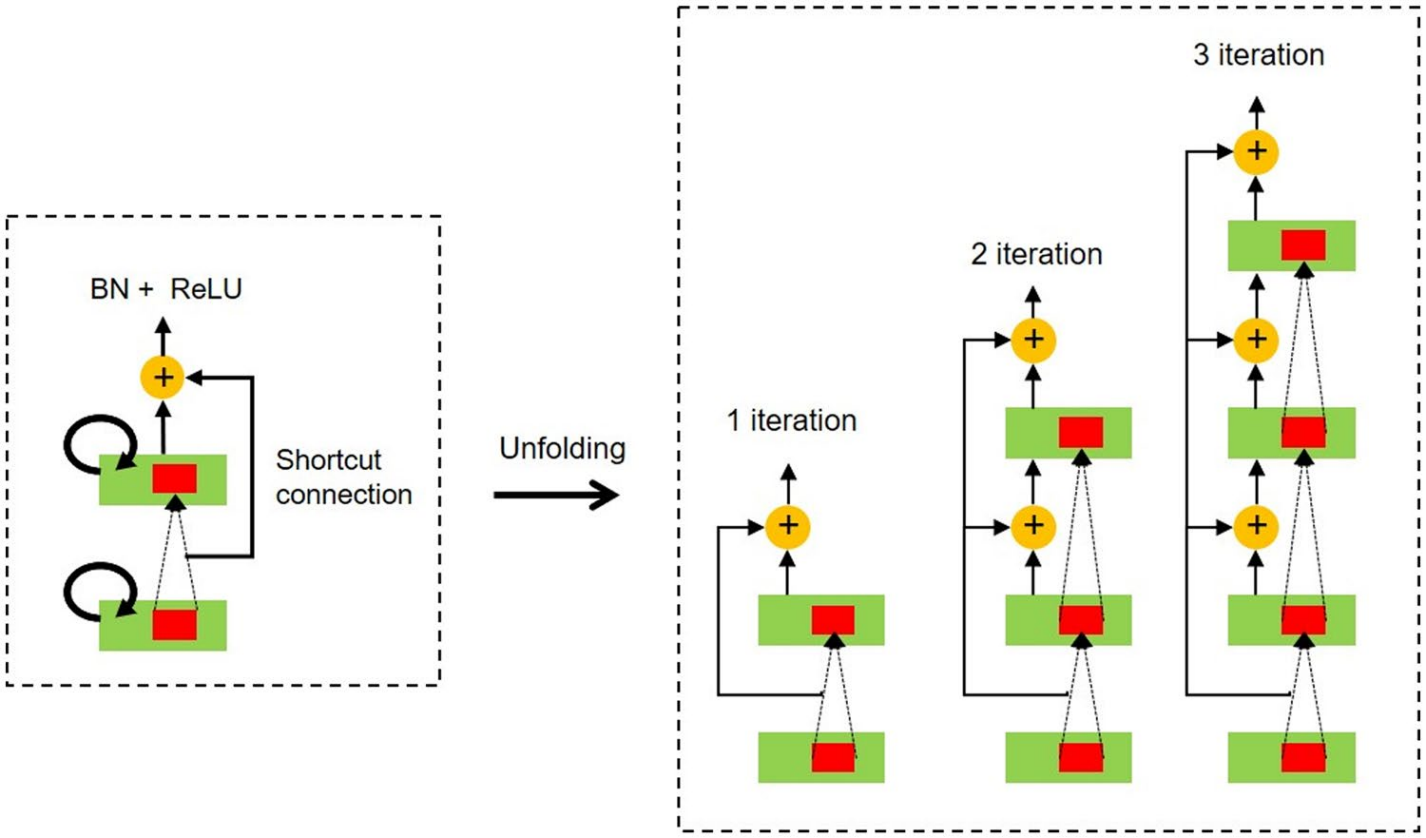
Recurrent residual block

Recurrent residual blocks are used in developing a more effective deeper model. Also, for better convergence, the effective feature accumulation is used in the R2U-Net [20, 21]. Due to the feature accumulation, it can make sure stronger and better feature expression in different time-steps. Figure 5 indicates the RRB.

### Training Deep Learning Model

We trained the model with Adam optimizer and dice loss function [22]. The learning rate is set to be 0.001 with keras callback function ReduceLROnPlateau, batch size of 10, and epochs of 200. A dice loss function is based on dice coefficient, which is a typical evaluating indicator commonly facilitated to verify the automated image segmentation model [23]. The dice loss function is defined as follows:

1 $${L}_{Dice}=1-\frac{2TP}{2TP+FP+FN}$$

True positive (TP), true negative (TN), false positive (FP), and false negative (FN) are calculated by comparing the ground truth and the pixel units, which are predicted results of the model.

### Post-processing

Some of the result images predicted by the model might show areas that are miss-detective or vague. Post-processing was done to get rid of this miss-detected area. If the area of domain per each bone is below a certain level, it was deemed miss-detected, then subsequently removed. Also, in the case of the void present in the bone area on predicted images, it was filled when the eight-direction pixel value was 1 based on the standard pixel.

### Vertebral Compression Ratio

**Fig. 6:**
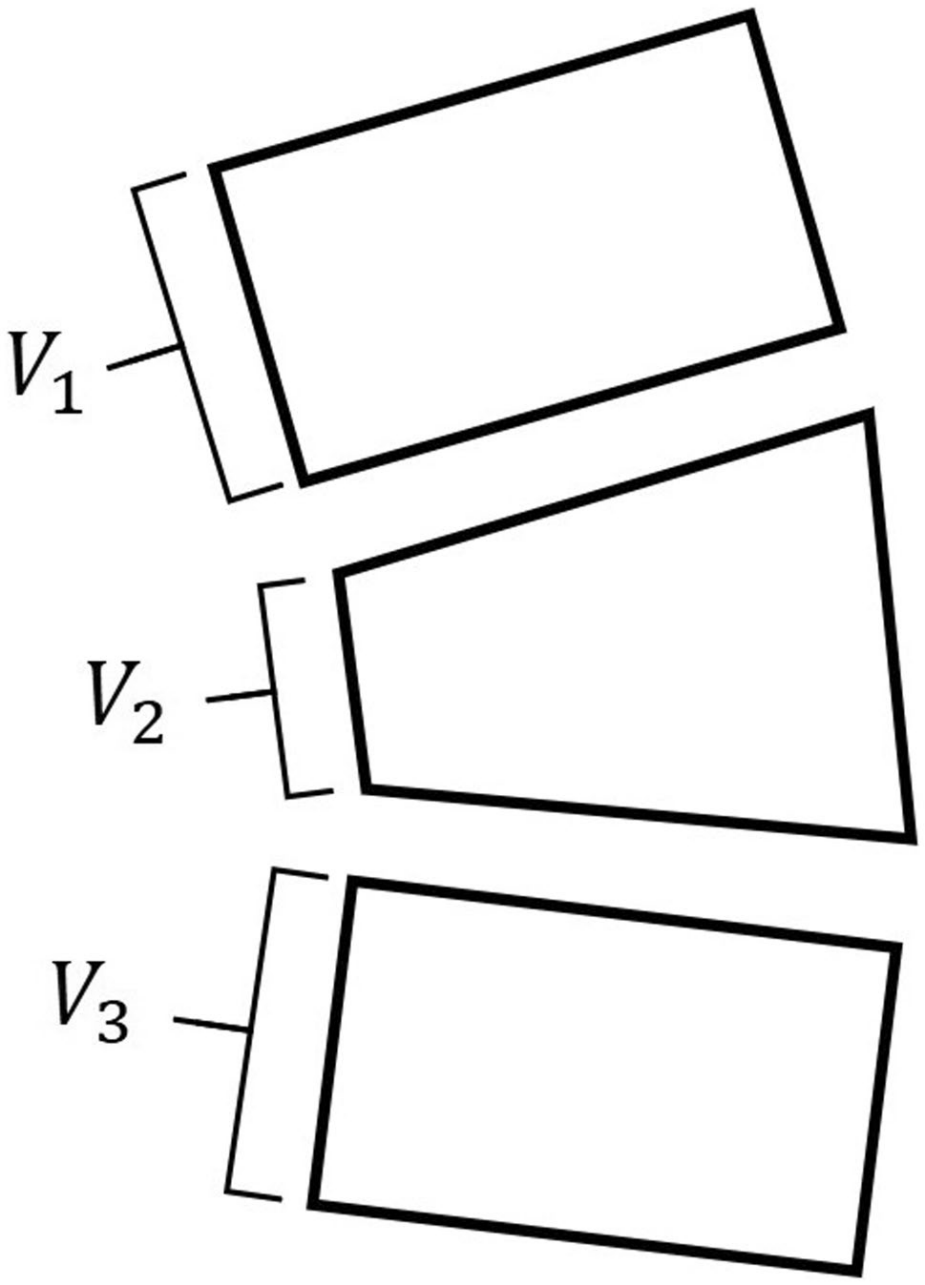
Method for calculating vertebral compression ratio

VCR, a ratio of abnormal to normal vertebral body height, is measured by the ratio of AVH and adjacent AVHs with respect to the vertebral body [8, 13, 24]. The extent of VCR determines no fracture, mild, moderate, or severe deformity of fracture and is a significant indicator to diagnose VCF [25, 26]. Figure 6 indicates how the VCR is measured and calculated.

2 $$VCR=[1-(\frac{{V}_{2}}{{(V}_{1}+{V}_{3})\times \frac{1}{2}})]\times 100)(\%)$$

## Result

**Fig. 7:**
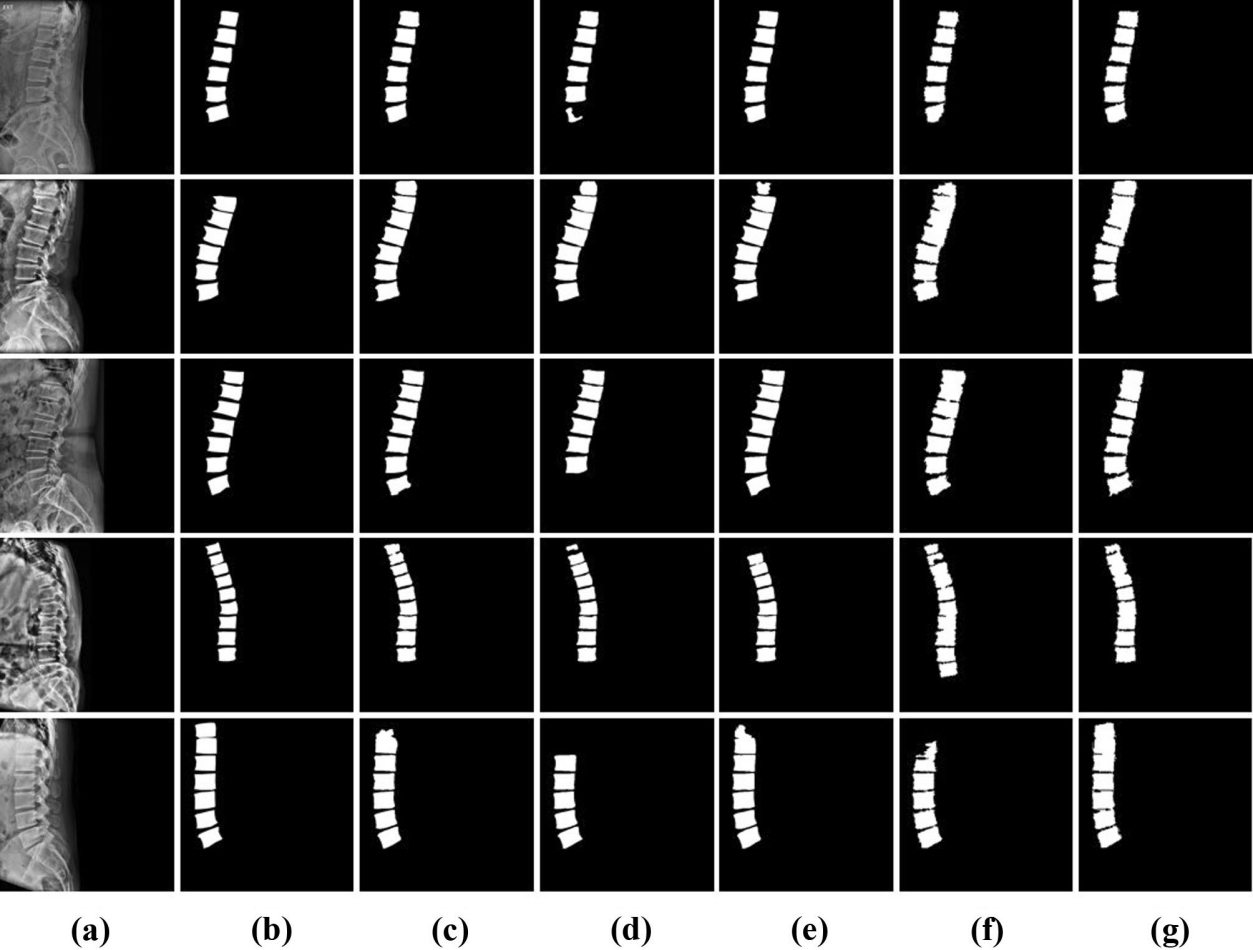
Images comparing between ground truth images and predict images. **a** Original images, **b** ground truth images, **c** U-Net, **d** SegNet, **e** R2U-Net, **f** E-Net, **g** MDR2U-Net

This study looked at the model for segmenting the vertebral images with the MDR2U-net model. Figure 7 indicates the comparisons between the images manually segmented by the spine specialists and the result images of the vertebral segmentation predicted by the models.

The trained model was verified via sensitivity, specificity, accuracy, and dice similarity coefficient. TP, TN, FP, and FN values were calculated by comparing vertebral area segmented by vertebral segmenting models. The values of sensitivity, specificity, accuracy, and dice similarity coefficient were calculated according to each formula.

3 $$dice\,similarity\,{coefficient}=\frac{2TP}{2TP+FP+FN}$$

**Fig. 8:**
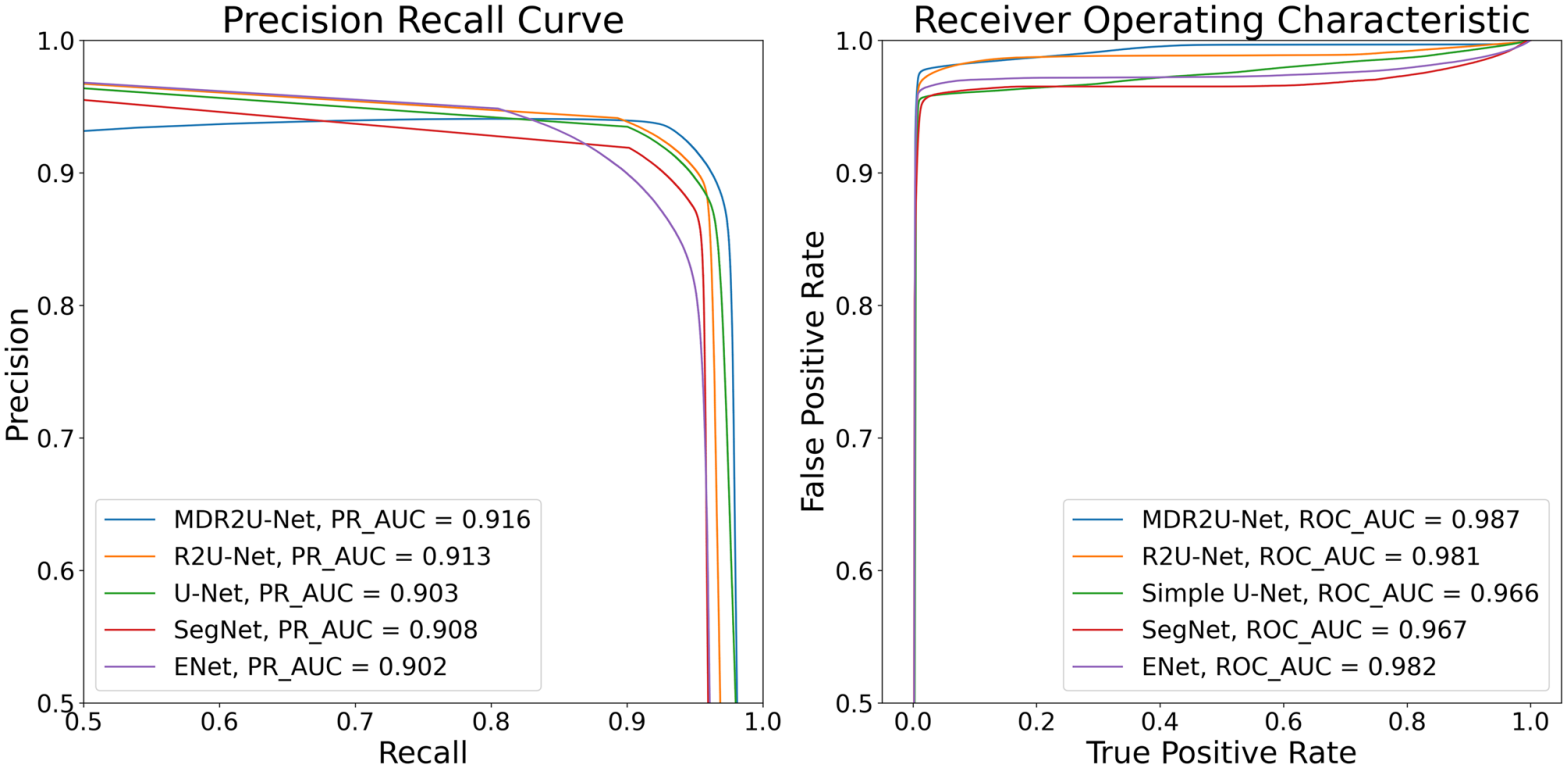
Receiver operating characteristic curve and precision recall curve

In evaluating the trained model performance, U-Net, R2U-Net, SegNet, and E-Net were used to compare with MDR2U-Net [19, 21]. Table 1 contains the comparison values of segmentation results with sensitivity, specificity, accuracy, dice similarity coefficient, and area under the curve (AUC) of receiver operation characteristic (ROC) and precision recall curve (PR). Also, Fig. 8 indicates ROC and PR curve.

**Table 1:** Comparison of segmentation performance between deep learning networks

Model	Sensitivity	Specificity	Accuracy	DSC	ROC_AUC	PR_AUC
U-Net	0.930 ± 5.012	0.994 ± 0.420	0.991 ± 0.485	0.919 ± 4.176	0.966	0.903
R2	0.933 ± 4.636	0.995 ± 0.329	0.991 ± 0.408	0.923 ± 3.383	0.981	0.913
SegNet	0.901 ± 6.221	0.993 ± 0.406	0.987 ± 0.506	0.890 ± 4.233	0.967	0.908
E-Net	0.926 ± 4.035	0.994 ± 0.399	0.990 ± 0.441	0.917 ± 3.600	0.982	0.902
Ours	0.937 ± 4.316	0.995 ± 0.349	0.992 ± 0.420	0.929 ± 3.386	0.987	0.916

**Fig. 9:**
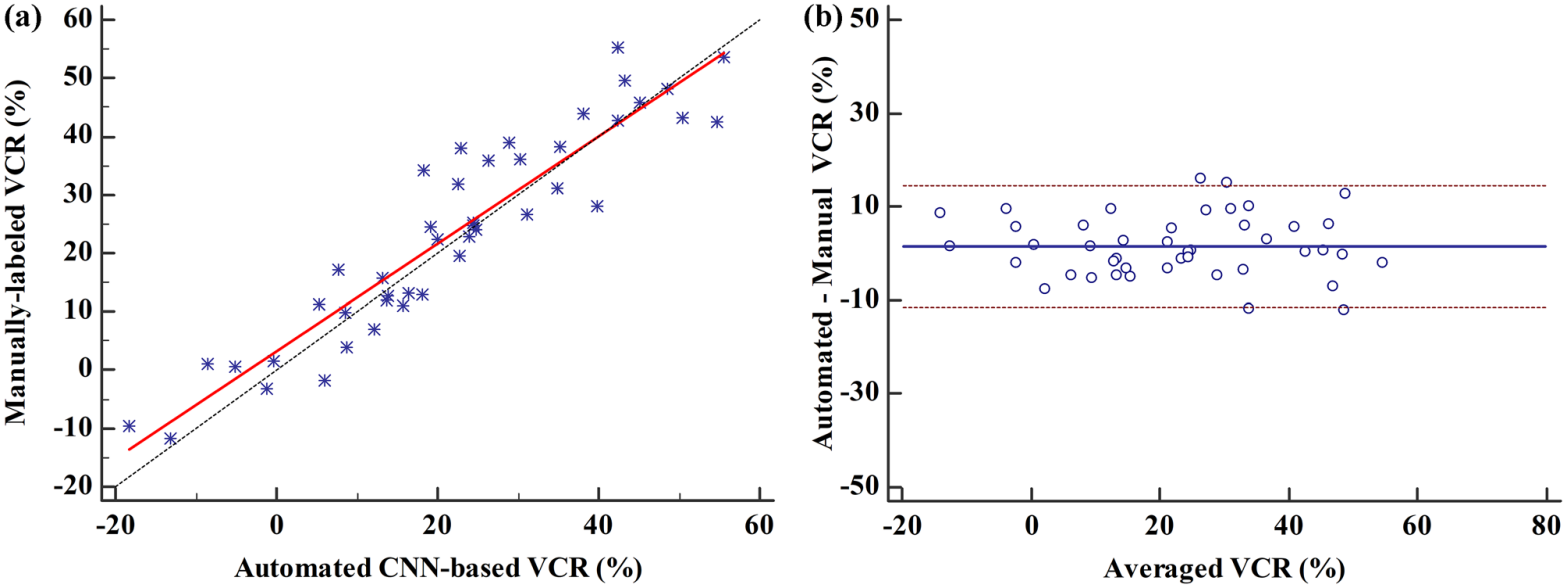
Comparison of manual VCR and measured VCR with predicted images. **a** Scatter plot, **b** Bland–Altman plot

VCR data that measured the segmented vertebral area through the trained model and the value of VCR data measured by the spine specialists based on the manually segmented vertebral area data were analyzed by correlation analysis. The correlation coefficient (*r*) was 0.929, which is a high value. It shows that VCR between manually measured by specialist and measured with predicted images by the model has no significant difference. Bland–Altman plot showed that most of the VCR values were in the area and within average ± 1.96, showing good equivalence. Figure 9 indicates the correlation between VCR manually measured by specialists and VCR measured with predicted images by model with the scatter plot and the Bland–Altman plot.

## Discussion

In this study, the model for vertebral segmentation using lateral spine X-ray images was trained and evaluated based on the results segmented manually by the specialists. We achieved the segmentation result with the sensitivity of 0.937, the specificity of 0.995, the accuracy of 0.992, and the dice similarity coefficient of 0.929.

Also, we performed comparative analysis on the VCR calculated based on the data on vertebral area manually segmented by the specialists and the VCR calculated by predicted data from trained vertebral segmentation model. Correlation analysis results showed no statistical significance on the two VCRs (*r* = 0.929). Bland Altman plot analysis indicates that the reliability between the VCRs measured by the specialists and predicted by the model, enabling us to confirm that the model for the vertebral segmentation using lateral spine X-ray images is useful in measuring VCR. As a result of the Bland–Altman plot analysis, the reliability between the compression ratio measured by the specialists and the compression ratio measured by the model predicted is high.

The vertebral segmentation model of this study led to accurate results, producing high reliability in VCRs. However, few data showed a substantial difference between VCR, the specialists measured, and the model predicted. We see that the reason behind this is due to the vertebral area not properly segmented in some data. Also, in terms of model training, the number of data of 339 patients may not be reliable for evaluating the performance of the model. To make up for this, fivefold cross-validation was used to increase the reliability for the performance of the model trained by few amounts of data [27].

In future studies, it is necessary to measure accurate VCRs by building a model with an outstanding performance on the vertebral segmentation, which can be done by improving the structure of the existing U-Net model, changing the training parameters, and finding the most optimal training parameter through experiments.

## Conclusion

In conclusion, training the model that segments the lateral spine X-ray images with deep learning led to excellent performance, and the VCRs measured with the data segmented by this model resulted in high reliability. Additional training and improvements of the model with plenty of data based on this would bring about accurate segmentation results on the vertebral area, and furthermore, the measurement of the precise and reliable VCRs would be guaranteed.
